# PcMab-47: Novel Antihuman Podocalyxin Monoclonal Antibody for Immunohistochemistry

**DOI:** 10.1089/mab.2017.0008

**Published:** 2017-04-01

**Authors:** Satoshi Ogasawara, Mika K. Kaneko, Shinji Yamada, Ryusuke Honma, Takuro Nakamura, Noriko Saidoh, Miyuki Yanaka, Kanae Yoshida, Yuki Fujii, Yukinari Kato

**Affiliations:** ^1^ Department of Regional Innovation, Tohoku University Graduate School of Medicine, Sendai, Japan.; ^2^ Department of Antibody Drug Development, Tohoku University Graduate School of Medicine, Sendai, Japan.; ^3^ New Industry Creation Hatchery Center, Tohoku University, Sendai, Japan.

**Keywords:** podocalyxin, PODXL, monoclonal antibody, immunohistochemistry

## Abstract

Podocalyxin (PODXL) is a CD34-related sialomucin and a well-known marker of embryonic stem cells. PODXL is expressed in many types of tumors including colorectal cancers, breast cancers, and brain tumors. Overexpression of PODXL is an independent predictor of progression, metastasis, and poor outcome. PODXL is also expressed in many normal cells such as renal podocytes and endothelial cells (ECs). However, high-sensitive and high-specific anti-PODXL monoclonal antibodies (mAbs) have not been established. Herein, we immunized mice with recombinant human PODXL, which was produced using LN229 glioblastoma cells. The anti-PODXL mAb, PcMab-47, reacted with endogenous PODXL-expressing cancer cell lines and normal cells independently of glycosylation in flow cytometry. Immunohistochemical analysis showed that PcMab-47 detected PODXL-expressing normal cells such as podocytes of kidney or ECs. Furthermore, PcMab-47 stained PODXL-expressing cancer cells of colon or breast cancers. These results suggest that PcMab-47 could be useful for investigating the expression and function of PODXL in cancers and normal tissues.

## Introduction

Podocalyxin (PODXL), which is also known as PCLP, MEP21, Gp200, Gp135, thrombomucin, GCTM2, TRA-1-60 antigen, or TRA-1-81 antigen, is a type I transmembrane protein with a molecular weight of 150,000–200,000.^(1–3)^ PODXL is expressed in normal tissues, including kidney, heart, pancreas, and breast tissues, as well as in neurons, ductal luminal cells, podocytes, endothelial cells (ECs), oviductal luminal cells, and mesothelial cells; PODXL also plays an important role in the development of tissues.^(4)^ PODXL is similar to CD34, which is known as a hematopoietic stem cell marker.^(5)^ A sialomucin, PODXL is highly glycosylated with *N*-glycan, *O*-glycan, and keratan sulfate.^(6)^ PODXL was first found in rat podocytes of the kidney,^(7)^ and its homologues have been reported in humans.^(8,9)^

PODXL has different functions in various tissues. PODXL acts as an adhesive molecule to bind to platelets or vascular endothelial cells (VECs)^(10,11)^; in contrast, PODXL functions as an antiadhesive molecule through its negatively charged mucin domain, including sialic acid and keratan sulfate, for the formation and maintenance of the filtration slits between the podocyte processes in the kidney.^(12)^ In addition, PODXL regulates cell morphology by associating with actin cytoskeletal proteins, such as Na^+^/H^+^ exchanger regulatory factor (NHERF)-1/2 and ezrin.^(13,14)^ PODXL is also known as one of the pluripotent stem cell markers. The antigen of the monoclonal antibodies (mAbs), TRA-1-60 and TRA-1-81, is keratan sulfate, which is attached to PODXL.^(3)^ The ligand of the recombinant N-terminal domain of the lectin BC2L-C from *Burkholderia cenocepacia* (rBC2LCN) is also reported as *O*-glycan of PODXL.^(15)^

PODXL is known as a diagnostic marker and prognostic indicator in several cancers, including brain tumors,^(6,16)^ prostate cancers,^(17)^ testicular tumors,^(2)^ renal cancers,^(18)^ oral cancers,^(19)^ thyroid cancers,^(20)^ bladder cancers,^(21)^ breast cancers,^(22–24)^ ovarian cancers,^(25)^ colorectal cancers,^(26–29)^ pancreatic cancers,^(30,31)^ and gastric cancers.^(32)^ The glycans on PODXL bind to P-/E-/L-selectin expressed on platelets, endothelium, and leukocytes, respectively.^(33–35)^ These interactions enhance the formation of platelet–tumor–leukocyte aggregates and tumor cell arrest in the microvasculature.^(36)^ Therefore, the overexpression of PODXL in cancer is a potential target for antibody therapy.

In this study, we established the anti-PODXL mAb, PcMab-47, for use in flow cytometry and immunohistochemistry.

## Materials and Methods

### Cell lines

LN229, Caco-2, MDA-MB-468, HEK-293T, Chinese hamster ovary (CHO)-K1, glycan-deficient CHO cell lines (Lec1, Lec2, and Lec8), and P3U1 were obtained from the American Type Culture Collection (Manassas, VA). Human VECs were purchased from Cambrex (Walkersville, MD). Lec13 was provided by Dr. Pamela Stanley.

LN229, Lec1, Lec2, Lec8, and Lec13 were transfected with PODXL plasmids, which included the ectodomain or full length of PODXL, using Lipofectamine 2000 (Thermo Fisher Scientific, Inc., Waltham, MA) according to the manufacturer's instructions. LN229/hPODXL-knockout (KO) cells (PDIS-13) were produced using CRISPR/Cas9 plasmids (Target ID: HS0000056763) against human PODXL (Sigma-Aldrich, St. Louis, MO).

The cell lines HEK-293T/GnT-1-KO (PDIS-12), HEK-293T/SLC35A1-KO (PDIS-22), HEK-293T/SLC35A2-KO (PDIS-18), and HEK-293T/GnT-1/SLC35A1/SLC35A2-KO (PDIS-20) were generated by transfecting TALEN or CRISPR/Cas9 plasmids, which target hsMgat1 (Wako Pure Chemical Industries Ltd., Osaka, Japan), SLC35A1 (Target ID: HS0000168432; Sigma-Aldrich), and SLC35A2 (Target ID: HS0000062603; Sigma-Aldrich), respectively, using a Gene Pulser Xcell electroporation system, and were screened using lectin profiling. These glycan-deficient cell lines are available from Cell Bank of Kato's Lab (www.med-tohoku-antibody.com/topics/001_paper_cell.htm) in Tohoku University (Miyagi, Japan).

CHO-K1, Lec1, Lec2, Lec8, Lec13, CHO-K1/PODXL, Lec1/PODXL, Lec2/PODXL, Lec8/PODXL, Lec13/PODXL, and P3U1 were cultured in RPMI 1640 medium, including l-glutamine (Nacalai Tesque, Inc., Kyoto, Japan). l-Proline (0.04 mg/mL) was added for Lec1, Lec2, Lec8, and Lec13. LN229, LN229/PODXL, LN229/ectodomain-PODXL, PDIS-13, HEK-293T, Caco-2, MDA-MB-468, PDIS-12, PDIS-22, PDIS-18, and PDIS-20 were cultured in Dulbecco's modified Eagle's medium, including l-glutamine (Nacalai Tesque, Inc.), supplemented with 10% heat-inactivated fetal bovine serum (FBS; Thermo Fisher Scientific, Inc.) at 37°C in a humidified atmosphere of 5% CO_2_ and 95% air. G418 (0.5 mg/mL; Wako Pure Chemical Industries Ltd.) was added for CHO-K1/PODXL, Lec1/PODXL, Lec2/PODXL, Lec8/PODXL, Lec13/PODXL, LN229/PODXL, and LN229/ectodomain-PODXL. VECs were cultured in EC medium EGM-2 MV supplemented with 5% FBS (Cambrex Corp.). Antibiotics, including 100 U/mL of penicillin, 100 μg/mL of streptomycin, and 25 μg/mL of amphotericin B (Nacalai Tesque, Inc.), were added to all media.

### Hybridoma production

Four-week-old female BALB/c mice (CLEA, Tokyo, Japan) were immunized by intraperitoneal (i.p.) injection of the purified ectodomain of human PODXL (100 μg) together with Imject Alum (Thermo Fisher Scientific, Inc.). After several additional immunizations, a booster i.p. injection of LN229/PODXL was given 2 days before the mice were euthanized by cervical dislocation, and spleen cells were harvested. The spleen cells were fused with P3U1 cells using PEG1500 (Roche Diagnostics, Indianapolis, IN). Hybridomas were grown in RPMI 1640 medium including l-glutamine with hypoxanthine, aminopterin, and thymidine selection medium supplement (Thermo Fisher Scientific, Inc.). Culture supernatants were screened using enzyme-linked immunosorbent assay (ELISA) for binding to the purified ectodomain of PODXL. Proteins were immobilized on Nunc Maxisorp 96-well immunoplates (Thermo Fisher Scientific, Inc.) at 1 μg/mL for 30 minutes. After blocking with 1% bovine serum albumin (BSA) in 0.05% Tween20/phosphate buffered saline (PBS; Nacalai Tesque, Inc.), the plates were incubated with culture supernatant followed by 1:2000 diluted peroxidase-conjugated antimouse IgG (Agilent Technologies, Inc., Santa Clara, CA). The enzymatic reaction was produced with a 1-Step Ultra TMB-ELISA (Thermo Fisher Scientific, Inc.). The optical density was measured at 655 nm using an iMark microplate reader (Bio-Rad Laboratories Inc., Hercules, CA).

### Flow cytometry

Cell lines were harvested by brief exposure to 0.25% Trypsin/1 mM EDTA (Nacalai Tesque, Inc.). After washing with 0.1% BSA in PBS, cells were treated with primary mAbs for 30 minutes at 4°C, followed by treatment with Oregon Green 488 goat antimouse IgG (Thermo Fisher Scientific, Inc.). Fluorescence data were collected using the Cell Analyzer EC800 (Sony Corp., Tokyo, Japan).

### Immunohistochemical analyses

Human normal tissues and cancer tissues were purchased from BioChain Institute, Inc. (Newark, CA) and US Biomax, Inc. (Rockville, MD), respectively. Four-micrometer-thick histological sections were deparaffinized in xylene and rehydrated. After the antigen retrieval procedure (autoclave using citrate buffer, pH 6.0; Agilent Technologies, Inc.), sections were incubated with 1 or 10 μg/mL of PcMab-47 for 1 or 3 hours at room temperature followed by treatment with Envision+ kit (Agilent Technologies, Inc.) for 30 minutes. Color was developed using 3, 3-diaminobenzidine tetrahydrochloride (Agilent Technologies, Inc.) for 5 minutes, and then the sections were counterstained with hematoxylin (Wako Pure Chemical Industries Ltd.).

## Results and Discussion

In our previous study, we developed the original technology for production of cancer-specific monoclonal antibodies (CasMabs) against membranous proteins.^(37)^ We successfully produced the antipodoplanin (PDPN) CasMab clone LpMab-2 that specifically recognizes cancer-type PDPN in tumor tissues and not in normal-type PDPN, which is expressed in lymphatic vessels.^(37)^ Moreover, the CasMab technology is useful for generating antiglycopeptide mAbs (GpMabs). We have produced the following anti-PDPN GpMabs: LpMab-3, LpMab-9, LpMab-12, LpMab-19, and LpMab-21.^(38–40)^ In addition, the CasMab technology can generate mAbs that bind to various novel epitopes of PDPN. We have produced the following anti-PDPN mAbs: LpMab-7, LpMab-10, and LpMab-17, which recognize novel epitopes.^(37,40–42)^ This study describes the production of sensitive and specific anti-PODXL mAbs using the CasMab technology.

Herein, we immunized mice with recombinant PODXL, which was purified from the culture supernatant of LN229/ectodomain-PODXL. A booster i.p. injection of LN229/PODXL was administered. The culture supernatants were screened using ELISA for binding to purified PODXL. As a second screening, we performed flow cytometry for reaction with LN229 and LN229/PODXL. A stronger reaction against LN229/PODXL than against LN229 was necessary, and one clone, PcMab-47 (mouse IgG_1_, kappa), was produced after limiting dilution.

**FIG. 1.**
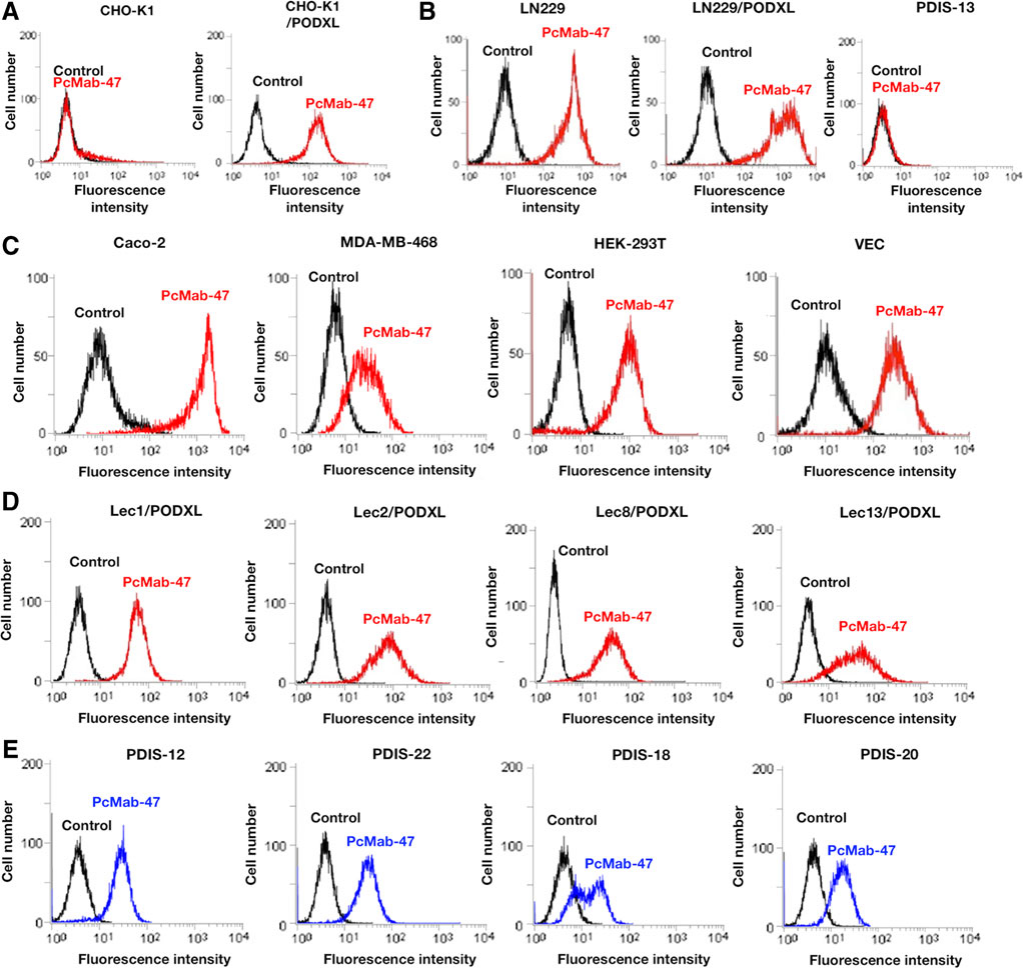
Specific detection of PODXL by PcMab-47 using flow cytometry. **(A)** CHO-K1 and CHO-K1/PODXL were treated with PcMab-47 (1 μg/mL; red) or control PBS (black) for 30 minutes at 4°C, followed by treatment with antimouse IgG-Oregon green. **(B)** LN229, LN229/PODXL, and PDIS-13 (LN229/PODXL-knockout cells) were treated with PcMab-47 (1 μg/mL; red) or control PBS (black) for 30 minutes at 4°C, followed by treatment with antimouse IgG-Oregon green. **(C)** Caco-2, MDA-MB-468, HEK-293T, and vascular endothelial cells were treated with PcMab-47 (1 μg/mL; red) or control PBS (black) for 30 minutes at 4°C, followed by treatment with antimouse IgG-Oregon green. **(D)** Lec1 (*N*-glycan-deficient CHO)/PODXL, Lec2 (sialic acid-deficient CHO)/PODXL, Lec8 (galactose-deficient CHO)/PODXL, and Lec13 (fucose-deficient CHO)/PODXL were treated with PcMab-47 (1 μg/mL; red) or control PBS (black) for 30 minutes at 4°C, followed by treatment with antimouse IgG-Oregon green. **(E)** PDIS-12 (*N*-glycan-deficient HEK-293T), PDIS-22 (sialic acid-deficient HEK-293T), PDIS-18 (galactose-deficient HEK-293T), and PDIS-20 (*N*-glycan/sialic acid/galactose-deficient HEK-293T) were treated with PcMab-47 (1 μg/mL; blue) or control PBS (black) for 30 minutes at 4°C, followed by treatment with antimouse IgG-Oregon green. Fluorescence data were collected using the Cell Analyzer EC800. PBS, phosphate buffered saline; PODXL, podocalyxin.

PcMab-47 reacted with CHO-K1/PODXL, and did not react with CHO (human PODXL-negative cell) in flow cytometry (Fig. 1A). PcMab-47 recognized endogenous PODXL, which is expressed in LN229 (a glioblastoma cell line), whereas it did not react with LN229/PODXL-KO cells (PDIS-13) (Fig. 1B), indicating that PcMab-47 is specific against PODXL. The reaction of PcMab-47 against LN229/PODXL was higher than that against LN229 (Fig. 1B).

PODXL is expressed in many cancers, such as breast cancers^(22–24)^ and colorectal cancers.^(26–29)^ We performed flow cytometry using several types of cancer cells and normal cells. PcMab-47 reacted with the cancer cell lines, such as Caco-2 (colon adenocarcinoma) and MDA-MB-468 (breast cancer) (Fig. 1C). PODXL is also expressed in normal tissues of kidney and ECs.^(4)^ PcMab-47 reacted with the normal cell lines, including HEK-293T (renal epithelial cell) and with vascular VECs (Fig. 1C). These results show the usefulness of PcMab-47 for detecting PODXL in flow cytometry.

We next investigated whether the epitope of PcMab-47 includes *N*-glycan or *O*-glycan because PODXL is highly glycosylated (Fig. 1D, E). As shown in Figure 1D, PcMab-47 reacted with all glycan-deficient PODXL-expressing CHO cells, specifically Lec1/PODXL (*N*-glycan-deficient), Lec2/PODXL (sialic acid-deficient), Lec8/PODXL (galactose-deficient), and Lec13/PODXL (fucose-deficient). Furthermore, PcMab-47 reacted with all glycan-deficient HEK-293T cells such as PDIS-12 (*N*-glycan-deficient), PDIS-22 (sialic acid-deficient), PDIS-18 (galactose-deficient), and PDIS-20 (*N*-glycan/sialic acid/galactose-deficient), indicating that glycans are not included in the PcMab-47 epitope (Fig. 1E).

**FIG. 2.**
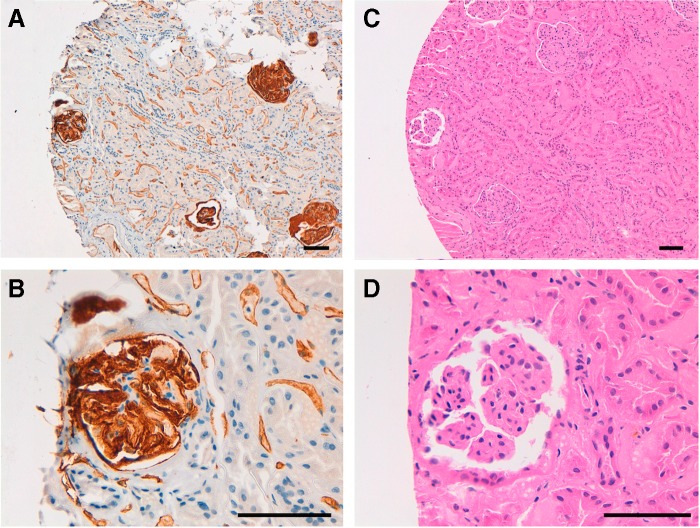
Immunohistochemical analysis of human kidney by PcMab-47. A section of kidney was incubated with 1 μg/mL of PcMab-47, followed by the Envision+ kit **(A, B)**. Color was developed using 3, 3-diaminobenzidine tetrahydrochloride, and counterstained with hematoxylin. Another section of kidney was also stained using hematoxylin and eosin **(C, D)**. Scale bar: 100 μm.

**FIG. 3.**
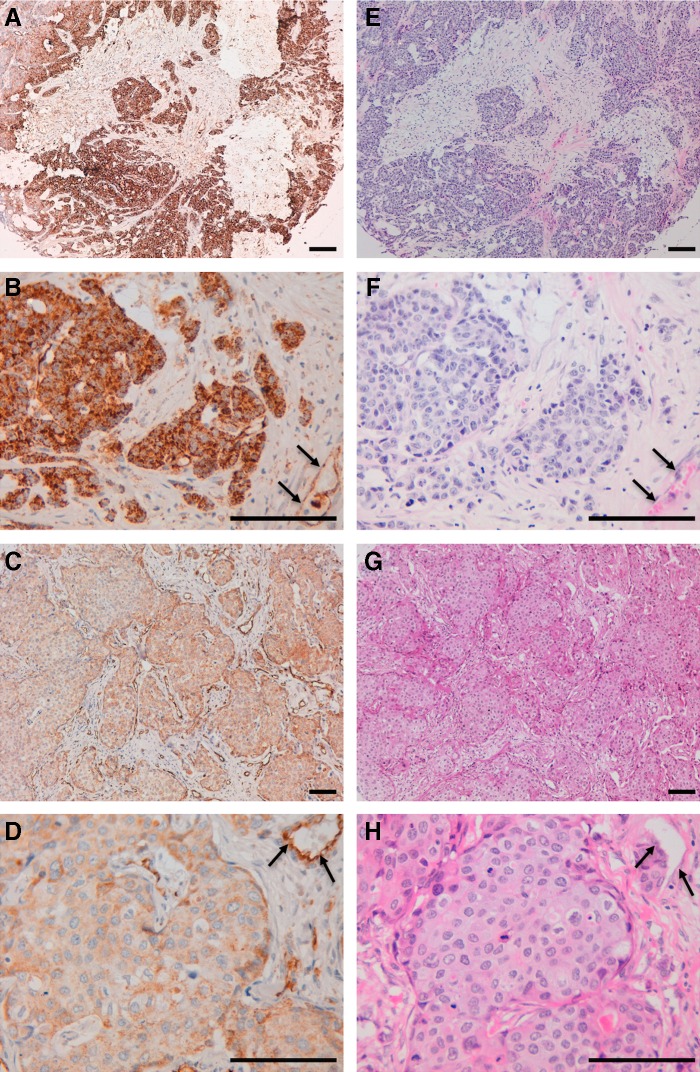
Immunohistochemical analysis of human cancers by PcMab-47. Sections of colon cancer **(A, B)** and breast cancer **(C, D)** were incubated with 10 μg/mL of PcMab-47, followed by the Envision+ kit. Color was developed using 3, 3-diaminobenzidine tetrahydrochloride and counterstained with hematoxylin. Colon cancer **(E, F)** and breast cancer **(G, H)** were also stained using hematoxylin and eosin. Arrows: endothelial cells. Scale bar: 100 μm.

Next, we investigated the immunohistochemical utility of PcMab-47 in normal tissues or cancers. As shown in Figure 2, PcMab-47 stained podocytes or ECs of kidney (Fig. 2A, B). PcMab-47 reacted with colon adenocarcinoma, in which membrane/cytoplasmic-staining pattern was observed (Fig. 3A, B). Strong expression of PODXL was also observed in ECs in colon adenocarcinoma (Fig. 3B). In contrast, weak expression of PODXL was observed in cancer cells, but stronger expression was detected in ECs in breast cancer (Fig. 3C, D). These results indicate that PcMab-47 is very useful for immunohistochemical detection of both cancer cells and normal cells that express PODXL.

Therefore, PcMab-47 may facilitate investigation of the expression and function of PODXL in cancer and normal tissues. Future investigations should include production of different epitope-possessing anti-PODXL mAbs to elucidate the function of PODXL.
